# Powder Activated Carbon Pretreatment of a Microfiltration Membrane for the Treatment of Surface Water

**DOI:** 10.3390/ijerph120911269

**Published:** 2015-09-10

**Authors:** Yali Song, Bingzhi Dong, Naiyun Gao, Xiaoyan Ma

**Affiliations:** 1 School of Civil Engineering and Architecture, Zhejiang University of Science and Technology, Hangzhou 310023, China; E-Mail: yali_song@sina.com; 2 College of Environmental Science and Engineering, Tongji University, Shanghai 200092, China; E-Mail: gaonaiyun@sina.com; 3 College of Civil Engineering and Architecture, Zhejiang University of Technology, Hangzhou 310032, China; E-Mail: mayaner620@163.com

**Keywords:** surface water, PAC, microfiltration, membrane flux, organic matter

## Abstract

This study focused on the effect of powder activated carbon (PAC) adsorption on microfiltration (MF) membrane performance. The results showed that PAC pretreatment offered high organic matter removal rates for both dissolved organic carbon (DOC) and ultraviolet absorbance at 254 nm (UV_254_) during 10–200 mg/L PAC dosage. The removal efficiencies of organic matter by MF membrane filtration decreased with the increase of organic matter removal rate by PAC adsorption. PAC mainly removed organic matter of about 3 kDa molecular weight (MW). MF membrane maintained more than 5 kDa MW organic matter on the membrane after PAC adsorption. The results of membrane filtration indicated that PAC pretreatment slightly promoted membrane flux, regardless of PAC dosage. It seems that the organic matter fouling membrane was concentrated in more than 3 kDa MW. PAC removed markedly less than 3 kDa MW organic matter and had less effect on more than 3 kDa organic matter. Thus, PAC cannot reduce membrane fouling.

## 1. Introduction

Membrane fouling is one of important issues of membrane application in water treatment [1,2]. Membrane fouling results in a decrease of membrane flux and poor performance. Some researches indicated that natural organic matter (NOM) is main foulant responsible for membrane fouling of microfiltration (MF) or ultrafiltration (UF) in drinking water [3,4,5]. In order to reduce organic matter fouled membrane, activated carbon adsorption may be an option [6,7,8].

Powder activated carbon (PAC) is widely used as an absorbent in water treatment. PAC has good adsorption performance for NOM, and thus it was adopted as pretreatment to reduce membrane fouling and improve membrane performance [9,10,11]. However, different conclusions were obtained for efficiencies of PAC in improving membrane performance. Some researches indicated that PAC can enhance membrane flux when PAC was maintained on the surface of membrane [12,13,14]. Mozia *et al.* proposed that the role of PAC suspended in a feed in the PAC/UF system is the adsorption of low molecular humic acid (HA). The membrane provides a physical barrier preventing the passage of the PAC, thus ensuring the retention of the organic compounds adsorbed on the PAC [15]. Matsui *et al.* found PAC resulted in the formation of larger floc particles, and the reversible TMP increase during each filtration cycle was mitigated by pretreatment with PAC [16]. Study on the effect of NOM in the coupled superfine-PAC/microfiltration membrane process showed that NOM proved to be the main culprit in membrane fouling and superfine-PAC actually decreased the fouling compared to NOM alone [17]. However, some researchers reported that the presence of PAC aggravated membrane fouling or had no effect on membrane flux [18,19,20]. Filloux *et al.* found that PAC particles were observed in the backwash water as they probably accumulated during the growth of the foulant layer [18]. Campinas *et al.* (2010) concluded that PAC neither promoted nor controlled the membrane reversible fouling [20].

In this study, PAC was used as pretreatment prior to MF membrane for treatment of surface water. The main purpose of the experiment is to investigate the effect of PAC on membrane performance, especially focused on removal rates of organic matter by PAC-MF process and improvement of membrane flux.

## 2. Experimental Methods

### 2.1. PAC Adsorption

Wooden PAC (Shanghai activated carbon Company, Shanghai, China) was used in this study. The grain size distribution is: >74 μm—71%, >44 μm—95%. Before use, PAC was homogenized by adding few deionized water. Special PAC dosage of 10, 50, 100, 200 mg/L was added into raw water, respectively. Surface water from Huangpu River (China) was used in this experiment. PAC was added in water sample and stirred for 1 min rapid mixing at 100 rpm and 30 min slow mixing at 30 rpm, and then water sample was filtrated by a 0.45 μm filter. Filtrated water sample was used for the following membrane filtration.

### 2.2. Microfiltration Filtration Procedure

**Figure 1:**
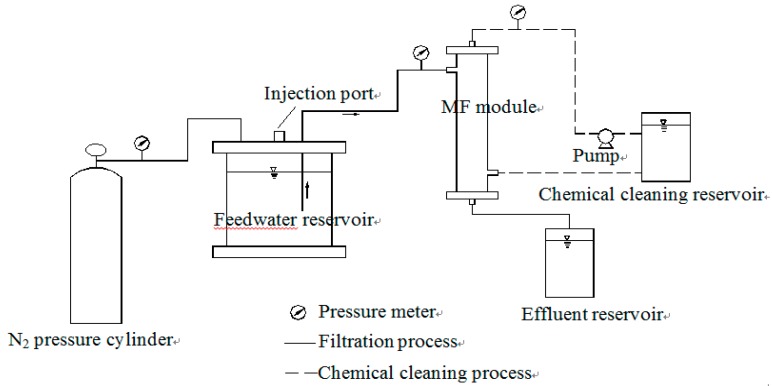
Schematic of microfiltration (MF) filtration apparatus.

MF treatment apparatus is presented in Figure 1. The membrane used in the experiment was a polyvinylidene fluoride (PVDF) hollow fiber MF membrane, with a nominal pore size of 0.1 μm and 75 cm^2^ surface areas (Toray, Tokyo, Japan). MF membrane was performed in dead—end and out—in operation mode. The MF experiment set-up concludes a N_2_ pressure cylinder, feed water reservoir, membrane module, effluent reservoir, chemical cleaning reservoir and peristaltic pump. A water sample in the feed water reservoir was fed by N_2_ into the membrane module. A magnetic stirrer at the bottom of feed water tank was used to completely mix the feed water. The filtration process was carried out under a pressure of 0.1 MPa. When 800 mL of the solution was filtrated, filtration finished and then chemical cleaning was carried out to restore membrane flux. The chemical cleaning process included two steps: 1% oxalic acid and 5 g/L sodium hypochlorite was pressed by peristaltic pump into MF membrane module and circularly cleaned membrane for 2 h, respectively.

### 2.3. Analytical Method

Molecular weight analysis was conducted by two methods: gel permeation chromatography (GPC) and UF membrane filtration. The GPC method is as follows [21]: A LC-10AD pump (Shimadzu, Kyoto, Japan), a SPD-20A UV detector (Shimadzu), a SCL-10A system controller (Shimadzu) and a G2500PWXL column (TSK, Shizuoka, Japan) consists of an HPLC system. Sodium sulfate was used as the moving phase. The MW distribution results were analyzed using the response (volt) data with elapsed time. In the UF membrane filtration method, 10, 7, 4, 2, 1 and 0.5 kDa UF membrane (Millipore, Boston, MA, America) was used to separate organic matter. The sample was filtered by a 0.45 μm membrane and then passed through the various UF membranes, respectively. Finally, seven organic fractions could be obtained as follows: <0.5 kDa, 0.5–1 kDa, 1–2 kDa, 2–4 kDa, 4–7 kDa, 7–10 kDa and >10 kDa. DOC concentration and UV_254_ absorbance were used to determine organic matter fraction amount.

The hydrophobic/hydrophilic fraction of organic matter was performed with resin method following the procedure as described by Carroll *et al*. [22]. Adsorbent resin (Supelite DAX-8, Supelco, Bellefonte, RA, America; Amberlite XAD-4, Sigma, Santa Clara, CA, America) was used to separate the organic substances into three groups: hydrophobic (HPO) fraction, transphilic (TPI) fraction and hydrophilic (HPI) fraction.

The concentration of organic matter was determined in terms of DOC and ultraviolet absorbance at 254 nm (UV_254_). All samples were filtered through a 0.45 µm membrane prior to DOC and UV_254_ measurement. DOC was analyzed in a TOC analyzer (Shimadzu TOC-VCPN). UV absorbance was measured in an UV spectrophotometer (DR5000, HACH, Loveland, CO, America).

## 3. Results

### 3.1. DOC and UV_254_ Removal Efficiencies of PAC and MF Process

**Figure 2:**
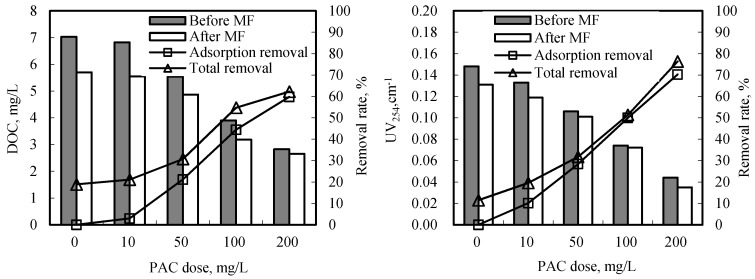
Efficiencies of DOC and ultraviolet absorbance at 254 nm (UV_254_) by powder activated carbon (PAC)/MF process.

After raw water adsorbed by PAC passing through MF membrane, the efficiencies of DOC and UV_254_ are shown in Figure 2. From the figure, PAC has a high removal rate of DOC. During PAC dosage of 10–200 mg/L, DOC removal rate increased from 3.0% to 59.8%. When the MF membrane filtrated the water sample after PAC adsorption, DOC removal of permeate increased steadily from 21.1% to 62.2%. However, DOC removal by MF membrane increased slightly with the increase of PAC dosage.

Compared to DOC, a high removal rate of UV_254_ was obtained. The UV absorbance removal increased from 10.1% to 70.3% with the increment of PAC dosage from 10 mg/L to 200 mg/L. Similar to the removal rate of DOC after MF filtration, the higher the PAC dosage, the lower the UV_254_ removal by the MF membrane. It is likely that PAC obtained a high removal of DOC and UV_254_ with high PAC dosage and few residual organic matters that can be removed by the membrane.

### 3.2. Effect of PAC on Molecular Weight Distribution

**Figure 3:**
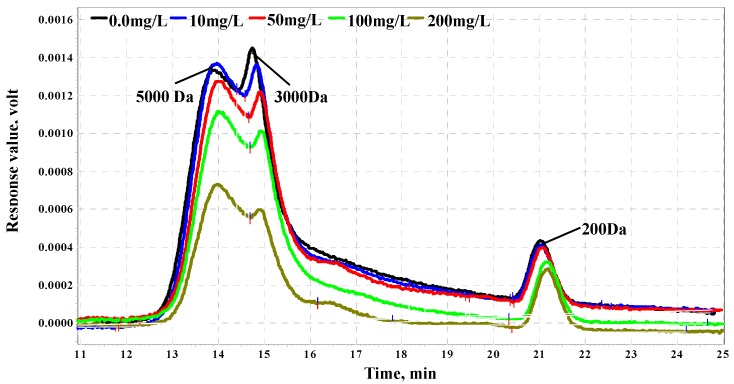
Effect of PAC on organics molecular weight distribution.

Organic matter molecular weight (MW) distributions with different PAC dosages are shown in Figure 3. With the increase of PAC dosage, the decrease of organic matter by about 3–5 kDa molecular weight is obvious, especially at about 3 kDa organic matter. When PAC dosage was increased to 200 mg/L, the molecular weight of 3–5 kDa dramatically decreased by the half of organic matter in raw water. With respect to about 0.2 kDa organics, PAC has no influence. Some research showed that PAC selectively adsorbed hydrophilic organic matter which has a low MW and is easilyadsorbed by PAC [23,24]. Thus, less than 3kDa MW organic matter was mostly removed by PAC. Liu *et al.* also found that PAC mainly removed 0.5-1 kDa and 1-3 kDa MW organic matter and has no effect on less than 0.5 kDa, which is similar to the result of organic matter MW by PAC adsorption in this study [25].

**Figure 4:**
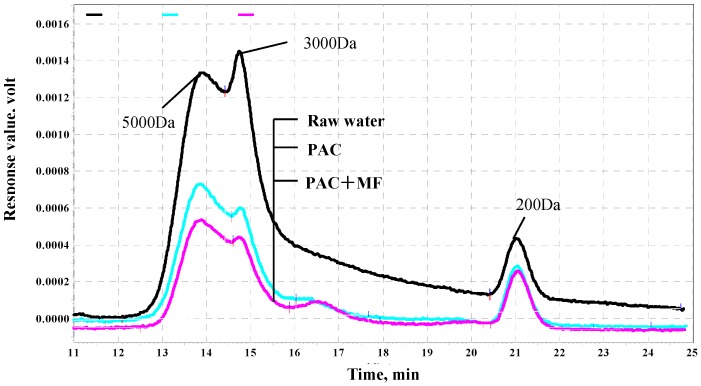
Effect of PAC and MF on organics molecular weight distribution.

The molecular weight distribution of organic matter by PAC/MF filtration was shown in Figure 4. From the figure, PAC has a significant effect on organic MW. Compared to PAC, MF membrane filtration has a slight effect on MW slight, which is similar to organic matter removal by PAC in Figure 2. For the MF membrane, about 5 kDa MW organic matter was removed by more than that of 3 kDa. It seems that the MF membrane was prone to removing higher MW organic matter.

### 3.3. Effect of PAC on Membrane Flux

**Figure 5:**
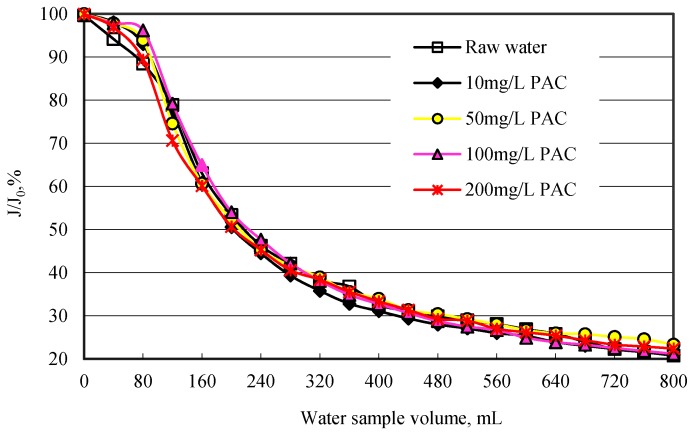
Membrane flux variation with different PAC dosage.

In this study, the ratio of membrane flux *(J*) to pure water flux (*J_0_*) was used to identify the membrane flux. Figure 5 shows the effect of PAC on membrane flux. As can be seen from Figure 5, PAC has almost no effect of enhancing the membrane flux, whatever dosage of PAC was added. Even though 200 mg/L PAC was added, the *J / J_0_* decreased to 22.4% after 800 mL water sample was filtered. When raw water was passed through the MF membrane without PAC adsorption, the *J / J_0_* decreased to 20.8%. The above results regarding the removal of organic matter by PAC indicated that PAC resulted in high removal of organic matter which is low MW organics. It is likely that the size of this low MW organic matter was smaller than that of membrane pore and did not deposit on membrane surface, and then consequently cannot result in membrane fouling. Obviously, the organic matter resulting in membrane fouling cannot be removed by PAC adsorption. Li *et al.* also found that PAC pretreatment would enhance the organic removal but at the same time increase membrane fouling. The fraction of NOM that is adsorbed by PAC might not induce much fouling on membranes [26].

## 4. Discussion

In the above study, PAC has a high removal rate for organic matter. However, PAC could not improve the membrane flux even at a very high dosage of 200 mg/L. MW of organic matter of raw water was concentrated on 2–7 kDa, and especially 3–5 kDa. Organic matter adsorbed by PAC concentrated on about 3 kDa MW. Obviously, PAC has little effect on higher MW organic matter.

**Figure 6:**
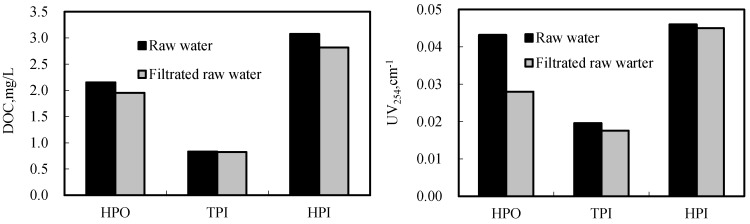
Change of organic hydrophobicity/hydrophilicity for raw water filtrated membrane filtration.

**Figure 7:**
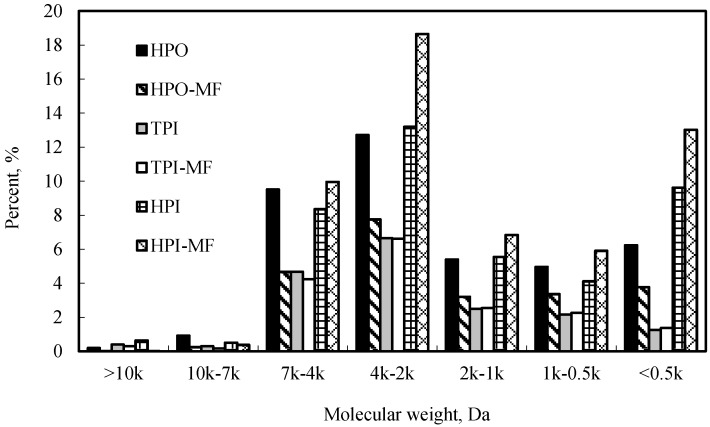
Change of organic matter molecular weight (MW) in raw water before and after membrane filtration.

In this study, changes of hydrophobicity/hydrophilicity of raw water after MF membrane filtration were shown in Figure 6. It can be seen that MF membrane mainly removed hydrophobic organic matter. Figure 7 showed the changes of hydrophobicity/hydrophilicity of different MW organic matter in raw water before and after membrane filtration. After membrane filtration, the proportion of hydrophobic organic matter markedly decreased, especially for 2–7 kDa MW organic matter. In contrast, the proportion of hydrophilic organic matter obviously increased. In this study, hydrophilic PVDF membrane was used. As a result, hydrophobic matter cannot pass through the membrane and stay on the membrane surface. Hydrophilic organic matter passed through membrane easily, and then the proportion of hydrophilic organic matter in the permeates increased markedly. It seems that the organic matter fouled membrane was hydrophobic organic matter which focused on 2–7 kDa MW. PAC only partly adsorbed 2–7 kDa MW organic matter and promoted membrane fouling slightly. This is the reason that PAC dramatically removed organic matter while enhanced membrane flux slightly.

## 5. Conclusions

In this study, PAC is used as pretreatment of MF membrane process in order to remove organic matter and reduce membrane fouling. PAC offers higher DOC and UV_254_ removal rates. During 20–200 mg/L PAC dosage, DOC removal rate of 3.0%-59.8% and UV_254_ removal rate of 10.1%–70.3% were obtained. However, organic matter removal rate by MF membrane was relatively low after PAC adsorption. The higher the PAC dosage, the lower the organic matter removal rate by MF. MW of organic matter in raw water was concentrated in 2–7 kDa, and especially 3-5 kDa. PAC removed 3 kDa MW organic matter more than in the case of 5 kDa MW. After PAC adsorption, membrane removed more 5 kDa MW than 3 kDa MW.

PAC improved membrane flux slightly, even at 200 mg/L PAC dosage, regardless of whether organic matter was dramatically removed by PAC. PAC adsorbed less than 3 kDa MW organic matter, which is not mainly responsible for membrane fouling. In Huangpu River raw water, the organic matter fouling membrane is hydrophobic higher MW organic matter. PAC could not markedly remove these organic matters, and thus it reduced membrane fouling slightly.
